# It’s all about connection: Determinants of social support and the influence on HIV treatment interruptions among people living with HIV in British Columbia, Canada

**DOI:** 10.1186/s12889-023-17416-7

**Published:** 2023-12-16

**Authors:** Clara Tam, Tim Wesseling, Lu Wang, Kate Salters, David M Moore, Nicole Dawydiuk, Julia Zhu, Sean Grieve, Brittany Bingham, Taylor McLinden, Robert Hogg, Rolando Barrios

**Affiliations:** 1 Epidemiology and Population Health Program, British Columbia Centre for Excellence in HIV/AIDS, St. Paul’s Hospital, 608-1081 Burrard Street, Vancouver, BC V6Z1Y6 Canada; 2 Faculty of Health Sciences, Simon Fraser University, Burnaby, Canada; 3 Faculty of Medicine, University of British Columbia, Vancouver, Canada; 4 Centre for Gender & Sexual Health Equity, University of British Columbia, Vancouver, Canada; 5 Indigenous Health, Vancouver Coastal Health, Vancouver, Canada

**Keywords:** HIV, Social support, Antiretroviral therapy, Treatment interruption, Adherence

## Abstract

**Background:**

Social support has previously been found to be associated with improved health outcomes of individuals managing chronic illnesses, including amongst people living with HIV (PLWH). For women and people who use injection drugs who continue to experience treatment disparities in comparison to other PLWH, social support may have potential in facilitating better treatment engagement and retention. In this analysis, we examined determinants of social support as measured by the Medical Outcomes Study – Social Support Survey (MOS-SSS) scale, and quantified the relationship between MOS-SSS and HIV treatment interruptions (TIs) among PLWH in British Columbia, Canada.

**Methods:**

Between January 2016 and September 2018, we used purposive sampling to enroll PLWH, 19 years of age or older living in British Columbia into the STOP HIV/AIDS Program Evaluation study. Participants completed a baseline survey at enrolment which included the MOS-SSS scale, where higher MOS-SSS scores indicated greater social support. Multivariable linear regression modeled the association between key explanatory variables and MOS-SSS scores, whereas multivariable logistic regression modeled the association between MOS-SSS scores and experiencing TIs while controlling for confounders.

**Results:**

Among 644 PLWH, we found that having a history of injection drug use more than 12 months ago but not within the last 12 months, self-identifying as Indigenous, and sexual activity in the last 12 months were positively associated with MOS-SSS, while being single, divorced, or dating (vs. married), experiences of lifetime violence, and diagnosis of a mental health disorder were inversely associated. In a separate multivariable model adjusted for gender, ethnicity, recent homelessness, sexual activity in the last 12 months, and recent injection drug use, we found that higher MOS-SSS scores, indicating more social support, were associated with a lower likelihood of HIV treatment interruptions (adjusted odds ratio: 0.90 per 10-unit increase, 95% confidence interval: 0.83, 0.99).

**Conclusions:**

Social support may be an important protective factor in ensuring HIV treatment continuity among PLWH. Future research should examine effective means to build social support among communities that have potential to promote increased treatment engagement.

**Supplementary Information:**

The online version contains supplementary material available at 10.1186/s12889-023-17416-7.

## Background

Social support has been explored in various health care contexts to better understand its effect on the health status of individuals. Higher social support has previously been found to be associated with improved health outcomes of those living with mental illness [1, 2], heart disease [3, 4], and diabetes [5] with individuals experiencing greater improvements in well-being [6, 7] and increased survival [8, 9]. In particular, social support has been shown to positively impact the health and well-being of individuals managing chronic illnesses [5, 10–12], including people living with HIV (PLWH). Among PLWH, higher social support has been found to be positively associated with higher measures of overall quality of life [13–16] and was also found to be associated with earlier HIV diagnosis [17], while lower social support was associated with depression [18, 19].

Definitions of social support vary widely but can be conceptualized and measured as two distinct concepts: structural support representing the diversity and number of supportive relationships (e.g., how many people do you know that you meet or talk to during a week); and functional support representing the quality and extent of supportive functions in these relationships (e.g., someone you can count on to listen to you when you need to talk) [20–22]. Functional social support, and the perception of available supports, has been shown to be a useful construct with identified impacts on medication adherence in a variety of settings [20–23]. In particular, functional social support among PLWH has the potential to encourage treatment adherence, with past studies finding individuals with higher social support having higher adherence to antiretroviral therapy (ART) [17, 24–27]. Along the HIV cascade of care, continuous ART use is critical to realizing the therapeutic and clinical benefits of modern treatment including reductions in HIV-related morbidity and mortality [28–34]. Consequently, since the advent of modern ART, there have been substantial improvements in life expectancy among PLWH [35–37]. Continuous use of ART has also been shown to eliminate onward HIV transmission, a concept known as Treatment as Prevention (TasP) [29, 38–42]. In British Columbia (BC), Canada, implementation of TasP as part of the global “undetectable equals untransmittable” (U = U) campaign [43–45] has been credited in part for a 66% decrease in new HIV diagnoses between 1996 and 2012 [29].

When gaps in continuous use of ART, also referred to as treatment interruptions (TIs), occur, it can lead to significant increases in HIV viral load and eventual declines in CD4 + cell counts which introduces risk of opportunistic infections, HIV transmission, and even death [29, 31, 46–48]. In Canada, individuals with a history of injection drug use (IDU), women, and racialized PLWH, have a higher likelihood of experiencing TIs, impacting life expectancy and other health outcomes [36, 48–51]. Within BC, studies have demonstrated that, even among those who are using ART, women [52–55], youth [48, 51, 56], people who use injection drugs (PWID) [48, 51, 57], and those with HCV-positive serostatus [48, 58] in particular, are more likely to experience TIs and have sub-optimal ART adherence. Despite the availability of publicly-funded ART, these treatment disparities highlight the potential role for targeted programs and supports to facilitate treatment uptake and retention amongst those who face greater socio-structural vulnerability.

Functional social supports may hold some promise in facilitating better treatment engagement [59] by addressing key disparities in treatment outcomes among sub-populations of PLWH. We designed a study to examine the determinants of functional social support and whether measures of social support were associated with treatment interruptions over time in a cohort of PLWH in British Columbia, Canada.

## Methods

### Study design

The STOP HIV/AIDS Program Evaluation (SHAPE) study is a longitudinal cohort of adult PLWH in BC, Canada. This study was designed to monitor health care encounters and therapeutic outcomes across BC as part of an evaluation of provincial HIV programs and interventions in the province and has been described at length previously [60]. To ensure research generated is based on community-identified needs and priorities, the SHAPE study is guided by a Steering Committee consisting of physicians, researchers, health service delivery decision makers, community organizations, community members with lived experience with HIV, and Indigenous community partners.

Enrolment into the SHAPE study took place between January 1, 2016 and September 1, 2018 across BC. Participants were eligible to participate in the SHAPE study if they were residents of the province of BC, had a confirmed HIV diagnosis, were at least 19 years of age or older at enrolment, able to provide informed consent, and were able to complete the survey tools in English [60]. SHAPE study participants were recruited using purposive sampling through posters and pamphlets distributed to community AIDS Service Organizations (ASOs), pharmacies, and clinics with the help of peer navigators, physicians, and health care workers, as well as through word-of-mouth in order to meet people where they are at. Recruitment quotas were established based on estimates of the proportion of PLWH found in the BC Centre for Excellence in HIV/AIDS (BC-CfE) centralized provincial Drug Treatment Program (DTP) registry in 2016 in order to maximize representativeness of the study population by age (age < 30 ~ 5%, age > 50 ~ 55%), gender (women ~ 20%), geographic location (residing in the Metropolitan Vancouver Area, including Vancouver the largest metropolitan area in BC ~ 75%), self-identifying as Indigenous (~ 15%), and key populations (gay, bisexual or other men who have sex with men [gbMSM] ~ 40%, PWID ~ 40%) [60, 61]. Some sampling quotas were relaxed in order to satisfy other quotas for participants who identified with several key characteristics (e.g. we oversampled those who identified as gbMSM to fill quotas regarding age). The BC-CfE DTP distributes ART at no cost to all medically eligible residents of BC, providing longitudinal health record data, including ART dispensing information, routine clinical data, and laboratory results of all individuals accessing ART in BC [62].

For this analysis, we utilized the SHAPE survey data collected at enrolment from January 2016 to September 2018, and examined treatment interruptions identified in the BC-CfE DTP that occurred in follow-up until December 2019. For inclusion in this analysis, participants were required to be on ART at time of enrolment, have at least 12 months of follow-up from the baseline interview date, and have complete responses for the 19-item Medical Outcomes Study-Social Support Survey (MOS-SSS) scale.

### Data collection

Participants provided written or oral consent prior to each survey with a peer research associate (PRA) or study staff member, and consented to data linkages with the BC-CfE centralized DTP registry, enabling linkage of participant survey responses to routine HIV-related clinical data. Individuals who could not be linked to the DTP were excluded from the study. Participants completed a baseline survey and were invited to complete two follow-up surveys approximately 18-months apart. Surveys were administered in-person or over the phone with PRAs, or self-administered online, depending on participant preference. The SHAPE survey collected data on sociodemographic characteristics including age, gender, geographic area of residence, education level, housing stability, and substance use, with additional validated scales measuring constructs such as food security [63] and access to social support [22]. The survey was developed in collaboration with study co-investigators and community members living with HIV, with input from the Steering Committee. Each survey took approximately 1.5 to 2 h to complete. Participants were provided with a $30 honorarium for each survey they completed, paid in cash or money order dependent on participant preference. Ethics approval was obtained from the University of British Columbia – Providence Health Care Research Institute ethics board (REB number: H15-01807).

### Measures

We conducted two analyses to examine social support (MOS-SSS) in our cohort. In our first analysis, select explanatory variables were considered as potential determinants of social support, with our main outcome variable of interest being social support. In our second analysis, the main explanatory variable of interest is MOS-SSS and the outcome is treatment interruptions.

#### Main variable of interest

The MOS-SSS is a validated instrument that measures multiple dimensions of perceived functional social support including tangible support, emotional-informational support, affectionate support and positive social interaction [22, 64, 65]. Respondents used a 5-point Likert scale to indicate how often each type of support is available when needed (response options range from “All of the time” to “None of the time”) (Supplementary File 1). An overall social support index for each SHAPE participant was calculated by summing individual item scores, averaging the scores for all 19 items to calculate the scale score, and then transforming scores to a range of 0 to 100, with higher scores indicating greater social support [22].

#### Determinants of social support analysis

The following explanatory variables were considered in association with the outcome of social support as a continuous variable. Participant age at enrolment, key populations (gbMSM, PWID), geographic location (Metropolitan Vancouver Area vs. outside Metropolitan Vancouver Area), and ethnicity (Caucasian, Indigenous, Asian, African, Black, Caribbean, Latin, Other) were collected from screening questions administered at the beginning of the survey. Participants were asked whether they self-identified as Indigenous in the baseline survey, with the term ‘Indigenous’ used here to collectively describe the Indigenous peoples of Canada inclusive of those who identify as First Nations, Inuit or Métis while acknowledging the diversity of cultures, languages, and traditions that exist among Indigenous peoples [66]. Additional explanatory variables including self-reported gender (male, female, other), sexual orientation (heterosexual, homosexual, other), relationship status (married/ common law/ steady partner, dating/ divorced/ separated/ single/ other), level of education (less than high school, completed high school, greater than high school), employment status (working, unemployed, other), housing stability (stable, neutral, not stable), homelessness in the last 12 months (yes vs. no), history of incarceration (ever vs. never), ever having a mental health diagnosis (yes vs. no), experiences of lifetime violence (defined as whether anyone has been violent to you in your lifetime, including physical abuse, emotional abuse, sexual abuse, someone controlling or restricting what you did, or other types of violence: yes vs. no), and history of IDU (in the past year vs. more than a year ago vs. never) were self-reported from the SHAPE survey questionnaire at the time of enrolment.

#### Social support and treatment interruptions analysis

We then examined the influence of social support as the main explanatory variable of interest using MOS-SSS scores, with HIV treatment interruption (TI) as the outcome variable. TI were defined as not receiving ART for 90 days or more based on prescription refill data found in the BC-CfE DTP [67–72]. TI were defined as 90 days past the stop date of the last prescription to factor in time where an individual may have delays in refilling their prescription but not experiencing a TI or if there is a delay by the pharmacy to notify the DTP regarding a missed prescription refill. TIs were grouped as a binary variable (yes or no), indicating whether participants had experienced one or more TIs during their follow-up time, from enrolment (completion of their first SHAPE survey) to December 2019.

### Statistical analysis

We performed descriptive statistics (n, %, Q1-Q3) of study participants at enrolment and used Kruskal-Wallis tests to examine variability in MOS-SSS scores across key explanatory variables. Univariable and multivariable linear regression were used to model the association between key explanatory variables and MOS-SSS scores as a continuous variable, with an assumption of linearity made between the explanatory and dependent variable. Explanatory variables were selected for potential inclusion in the multivariable model based on a priori knowledge from previous literature, univariable associations, and consultation with living experience. Variables that were highly correlated were not included in the model to reduce selection bias. Model selections were conducted using a backward stepwise technique which is based on both Akaike information criterion (AIC) and Type III p-values [73, 74]. The variable with the highest Type III p-value was dropped at each step of the selection process until the model reached the lowest AIC, with a lower AIC indicating better model fit.

Univariable and multivariable logistic regression was then used to model the association between MOS-SSS scores and TIs while controlling for confounding variables. MOS-SSS scores were modelled as a continuous variable with 10-unit increments to assist with the interpretation of increases in MOS-SSS scores due to the large range (possible scores from 0 to 100). Confounders considered for inclusion were based on previous literature. Confounders were selected for inclusion in the final model using a backward stepwise selection approach, which used the relative change in coefficients of the MOS-SSS score as a criterion, until the minimum change from the full model exceeded 5% [75–78]. We also examined the association between MOS-SSS scores and the number of TIs. We performed descriptive statistics (median, Q1-Q3) and conducted Wilcoxon rank-sum tests to examine variability in MOS-SSS scores with 0, 1, and > 1 TIs. All analyses were conducted in SAS version 9.4 (Cary, North Carolina, USA).

## Results

Of 644 participants who completed the baseline survey, 605 study participants were included in this analysis. Of the 39 participants excluded from the analysis, four were excluded due to having incomplete MOS-SSS scale items, 25 for having less than one year of follow-up in the BC-CfE DTP, three for never having any ART prescription recorded in the DTP, and seven for not receiving ART at enrolment.

Among 605 participants included in the analytic sample, 127 (21.0%) identified as female, and 320 (52.9%) were 50 years of age or older at the time of study enrolment. With respect to key populations, 365 (60.3%) self-identified as gbMSM, and 157 (26.0%) self-identified as PWID (see Table 1 bivariate analysis). The majority of participants resided in Metropolitan Vancouver Area (n = 424, 70.1%), while the remainder resided outside of the Metropolitan Vancouver Area (n = 181, 29.9%), 471 (77.9%) completed high school education or greater, and 400 (66.1%) were not currently in a relationship at enrolment. More than 50% of participants felt their housing situation was stable (n = 470, 77.7%) while 81 (13.4%) participants reported experiencing homelessness in the last year, 404 (66.8%) had been diagnosed with a mental health disorder, 206 (34.0%) had any previous experiences of incarceration, 459 (77.3%) experienced lifetime violence, and 460 (76.2%) were diagnosed with other illnesses (including Hepatitis C, diabetes, cancer, asthma, and high blood pressure through self-report). Participants had been receiving ART from the BC-CfE DTP for a median length of time of 9.4 years (Q1-Q3: 5.5–17.7).

**Table 1:** Descriptive of sociodemographic characteristics by Medical Outcomes Study – Social Support Survey (MOS-SSS) scale score

Variable	Overall (N = 605) N (%)	MOS-SSS Score (N = 605) Median (Q1-Q3)	P-value
**Age at interview**			0.509
Less than 40 40 to 49 50 to 59 60 or more	118 (19.5) 167 (27.6) 219 (36.2) 101 (16.7)	57.9 (39.5–82.9) 68.4 (43.4–86.8) 65.8 (42.1–86.8) 64.5 (47.4–84.2)	
**Gender**			0.275
Male Female Other	467 (77.2) 127 (21.0) 11 (1.8)	63.2 (40.8–85.5) 68.4 (46.1–86.8) 68.4 (60.5–80.3)	
**Sexual orientation**			0.795
Heterosexual Homosexual Other	213 (35.2) 281 (46.4) 111 (18.3)	63.2 (46.1–85.5) 64.5 (40.8–85.5) 64.5 (40.8–84.2)	
**Current relationship status**			**< 0.001**
Married/Common Law/Steady Partner Single/Dating/ Divorced/ Separated/ Other	205 (33.9) 400 (66.1)	82.9 (63.2–94.7) 55.3 (33.6–74.3)	
**Key populations**			0.101
MSM only IDU only Both MSM and IDU Neither MSM nor IDU	326 (53.9) 118 (19.5) 39 (6.4) 122 (20.2)	65.8 (43.4–85.5) 67.1 (43.4–90.8) 52.6 (31.6–75.0) 62.5 (44.7–84.2)	
**Geographic location**			0.935
Metropolitan Vancouver Area^a^ Outside Metropolitan Vancouver Area	424 (70.1) 181 (29.9)	64.5 (42.1–85.5) 63.2 (44.7–85.5)	
**Ethnicity**			0.382
Caucasian Indigenous Asian/ African/ Black/ Caribbean/ Latin Other	425 (70.2) 90 (14.9) 46 (7.6) 44(7.3)	64.5 (40.8–86.8) 68.4 (48.7–82.9) 54.6 (32.9–80.3) 64.5 (46.7–86.2)	
**Highest level of education**			0.263
Less than high school Completed high school Completed greater than high school	134 (22.1) 176 (29.1) 295 (48.8)	66.4 (43.4–89.5) 66.4 (45.4–84.2) 63.2 (39.5–84.2)	
**Employment status**			0.071
Working Unemployed Other	221 (36.5) 240 (39.7) 144 (23.8)	68.4 (48.7–86.8) 59.2 (39.5–82.2) 67.1 (43.4–87.5)	
**Incarceration ever**			
No	399 (66.0)	64.5 (43.4–84.2)	
Yes	206 (34.0)	63.8 (40.8–90.8)	0.548
**Homelessness in the last 12 months**			
No	524 (86.6)	65.8 (43.4–85.5)	
Yes	81 (13.4)	55.3 (38.2–78.9)	0.053
**Housing stability** ^**b**^			**< 0.001**
Stable Neutral Not stable	470 (77.7) 50 (8.3) 85 (14.0)	68.4 (47.4–88.2) 56.6 (34.2–82.9) 43.4 (26.3–64.5)	
**Mental health disorder diagnosis ever**			
No	201 (33.2)	69.7 (51.3–88.2)	
Yes	404 (66.8)	61.2 (39.5–84.2)	**0.002**
**Recent hazardous alcohol use**			
No	364 (60.2)	62.5 (40.8–84.2)	
Yes	241 (39.8)	65.8 (46.1–86.8)	0.164
**Lifetime violence** (n = 594)			
No	135 (22.7)	71.1 (48.7–90.8)	
Yes	459 (77.3)	63.2 (40.8–84.2)	**0.011**
**Sexual activity in the last 12 months** (n = 590)			
No	211 (35.8)	56.6 (34.2–84.2)	
Yes	379 (64.2)	68.4 (48.7–86.8)	**0.001**
**Current tobacco smoker**			0.445
Never a smoker Not current (ex smoker) Current smoker	171 (28.3) 149 (24.6) 285 (47.1)	67.1 (48.7–84.2) 64.5 (42.1–85.5) 61.8 (39.5–85.5)	
**History of IDU**			
Never Yes but not in the past year	352 (58.2) 132 (21.8)	65.8 (47.4–84.2) 68.4 (44.7–89.5)	
Yes in the past year	121 (20.0)	56.6 (32.9–75.0)	**0.018**
**Treatment interruptions**			**0.031**
No Yes	508 (84.0) 97 (16.0)	65.8 (44.7–85.5) 55.3 (38.2–78.9)	

Bold text refers to p-values < 0.05. Kruskal Wallis test was used to determine p-values of explanatory variables

MSM: men who have sex with men

IDU: injection drug use

^a^ Metropolitan Vancouver Area includes Vancouver, the largest metropolitan area in BC and the Vancouver Coastal and Fraser Health Authorities

^b^ Housing stability is defined as whether an individual feels their housing situation is stable (i.e. housing is safe, secure and affordable)

The median MOS-SSS score among all study participants was 64.5 (Q1-Q3: 42.1–85.5). We did not find statistically significant variations in MOS-SSS scores by age, gender, sexual orientation, key population membership, geographic location, or ethnicity (p-value > 0.05) (see Table 1). However, higher MOS-SSS scores were found among participants who were married (vs. single/ divorced/ separated, median 82.9 vs. 55.3, p-value < 0.001), experienced stable housing (vs. unstable, 68.4 vs. 43.4, p-value < 0.001), had never been diagnosed with a mental health disorder (69.7 vs. 61.2, p-value = 0.002), never experienced lifetime violence (71.1 vs. 63.2, p-value = 0.011), reported sexual activity in the last 12 months (71.1 vs. 63.2, p-value = 0.001), and those with no recent injection substance use (never 65.8 vs. not within the last year 68.4 vs. yes in the last year 56.6, p-value = 0.018). Furthermore, individuals who did not experience treatment interruptions within the study period reported higher social support scores (65.8 vs. 55.3, p-value = 0.031).

Table 2 shows our linear regression analysis of factors associated with MOS-SSS scores. In our final multivariable model, reporting a history of IDU greater than 12 months ago but not within the last 12 months (vs. no history of IDU; unstandardized beta coefficient [B] = 6.58, 95% confidence interval [CI] 1.43, 11.73), identifying as Indigenous (vs. Caucasian; B = 7.40, 95% CI 1.47, 13.32) and reporting sexual activity in the past 12 months (B = 4.84, 95% CI 0.42, 9.26) were associated with higher MOS-SSS scores. Being single, divorced or dating (vs. in a relationship; B = -21.23, 95% CI -25.60, -16.85), experiencing lifetime violence (B = -5.73, 95% CI -10.74, -0.73), or reporting being diagnosed with a mental health disorder (B = -4.17, 95% CI -8.47, 0.13) were associated with lower MOS-SSS scores.

**Table 2:** Univariable and multivariable linear regression of key sociodemographic characteristics and MOS-SSS scale score

	Univariable Linear Regressions		Multivariable Linear Regression	
**Variables**	B coefficient^a^ (95% CI)	P-value	B coefficient^a^ (95% CI)	P-value
**Age at interview**				
Less than 40	Ref		Not selected^b^	
40 to 49	4.57 (-1.78, 10.91)	0.159		
50 to 59	3.09 (-2.93, 9.12)	0.315		
60 or more	2.30 (-4.85, 9.46)	0.528		
**Gender**				
Male	Ref			
Female	4.58 (-0.69, 9.86)	0.089	Not selected^b^	
Other	5.04 (-11.05, 21.13)	0.539		
**Sex orientation**				
Heterosexual	Ref			
Homosexual	-1.07 (-5.87, 3.73)	0.662		
Other	-2.36 (-8.55, 3.82)	0.454		
**Current relationship status**				
Married/Common Law/Steady Partner	Ref		Ref	
Single/Dating/Divorced/ Separated/Other	-22.25 (-26.43, -18.07)	**< 0.001**	-21.23 (-25.60, -16.85)	**< 0.001**
**Key populations**				
MSM only	Ref			
IDU only	2.00 (-3.65, 7.65)	0.488		
Neither MSM nor IDU	-2.34 (-7.93, 3.24)	0.411		
Both MSM and IDU	-9.21 (-18.13, -0.29)	**0.043**		
**Geographic location**				
Metropolitan Vancouver Area^c^	Ref		Not selected^b^	
Outside Metropolitan Vancouver Area	− 0.039 (-5.08, 4.31)	0.871		
**Ethnicity**				
Caucasian	Ref		Ref	
Indigenous	4.65 (-1.46, 10.77)	0.136	7.40 (1.47, 13.32)	**0.015**
Asian/African/Black/Caribbean/Latin	-4.01 (-12.19, 4.17)	0.337	-6.07 (-13.77, 1.64)	0.123
Other	-0.70 (-9.05, 7.65)	0.869	2.50 (-5.21, 10.22)	0.525
**Highest education**				
Less than high school	Ref			
Completed high school	-1.65 (-7.70, 4.40)	0.593		
Completed greater than high school	-4.38 (-9.88, 1.11)	0.118		
**Employment status**				
Working	Ref		Not selected^b^	
Unemployed	-5.04 (-9.96, -0.13)	**0.044**		
Other	-1.26 (-6.90, 4.38)	0.662		
**Incarceration ever**				
No Yes	Ref 1.08 (-3.45, 5.62)	0.640	Not selected^b^	
**Homelessness in the last 12 months**				
No Yes	Ref -6.35 (-12.64, -0.05)	**0.048**	Not selected^b^	
**Housing stability** ^**d**^				
Stable	Ref			
Neutral	-8.26 (-15.89, -0.63)	**0.034**		
Not stable	-18.43 (-24.47, -12.38)	**< 0.001**		
**Mental health diagnosis ever**				
No Yes	Ref -7.03 (-11.56, -2.50)	**0.002**	Ref -4.17 (-8.47, 0.13)	0.057
**Recent hazardous alcohol use**				
No	Ref			
Yes	3.61 (-0.77, 8.00)	0.106		
**Lifetime violence** (n = 594)				
No Yes	Ref -6.22 (-11.40, -1.05)	**0.018**	Ref -5.73 (-10.74, -0.73)	**0.025**
**Sexual activity in the last 12 months** (n = 590)				
No Yes	Ref 8.25 (3.75, 12.75)	**0.000**	Ref 4.84 (0.42, 9.26)	**0.032**
**History of IDU**				
Never	Ref		Ref	
Yes but more than a year ago	2.69 (-2.67, 8.05)	0.326	6.58 (1.43, 11.73)	**0.012**
Yes in the past year	-6.68 (-12.21, -1.15)	**0.018**	-2.07 (-7.47, 3.34)	0.453

Bold text refers to p-values < 0.05

CI: confidence interval

MSM: men who have sex with men

IDU: injection drug use

^a^B coefficient is used to represent the unstandardized beta coefficient

^b^Explanatory variables were selected for potential inclusion in the multivariable model based on univariable associations and a priori knowledge from previous literature and consultation with living experience. Model selections were conducted using a backward stepwise technique which is based on Type III p-values and Akaike information criterion (AIC). The variable with the highest Type III p-value was dropped at each step of the selection process until the model reached the lowest AIC, with a lower AIC indicating better model fit

^c^Metropolitan Vancouver Area includes Vancouver, the largest metropolitan area in BC and the Vancouver Coastal and Fraser Health Authorities

^d^Housing stability is defined as whether an individual feels their housing situation is stable (i.e. housing is safe, secure and affordable)

A total of 97 (16.0%) participants experienced at least one TI event over the study period, and 24 (4.0%) participants experienced more than one TI, with a median follow-up time in the study of 3.2 years (Q1-Q3: 2.4–3.7) ending on December 2019. The median length of TI was 128 days (Q1-Q3: 106–268). The median MOS-SSS score for participants who experienced at least one TI was 55.3 (Q1-Q3: 38.2–78.9) compared to those who did not experience a TI was 65.8 (Q1-Q3: 44.7–85.5) (p-value = 0.03). The median MOS-SSS score for participants who experienced only one TI was 60.5 (Q1-Q3: 38.2–84.2) and more than one TI was 47.4 (Q1-Q3: 36.2–68.4) (p-value = 0.13).

In our multivariable logistic regression model, higher MOS-SSS scores (per 10-unit increase) were associated with decreased odds of TIs (adjusted odds ratio [aOR] 0.90 per 10-unit increase, 95% CI 0.83, 0.99) while controlling for confounders (see Table 3). Confounders selected for inclusion in the model were gender, ethnicity, experiences of homelessness in the past year, sexual activity in the last 12 months, and those who reported any IDU ever. Age, employment status, history of incarceration, and ever having been diagnosed with a mental health disorder were not selected for in the model after a change-in-estimates confounder selection approach.

**Table 3:** Multivariable logistic regression of the association between MOS-SSS and experiencing ≥1 TI, controlling for confounders

**Variables**	Univariable Logistic Regressions		Multivariable Logistic Regression	
	Odds ratio (95% CI)	P-value	Adjusted odds ratio (95% CI)	P-value
**MOS-SSS score** (per ten unit increase)^**b**^	0.92 (0.85, 0.99)	**0.031**	0.90 (0.83, 0.99)	**0.022**
**Age at interview**				
Less than 40	Ref			
40 to 49	0.70 (0.40, 1.22)	0.208	Not selected^a^	
50 to 59	0.41 (0.23, 0.74)	**0.003**		
60 or more	0.25 (0.11, 0.58)	**0.001**		
**Gender**				
Male	Ref		Ref	
Female	2.58 (1.61, 4.15)	**< 0.001**	1.75 (1.01, 3.03)	**0.045**
Other	1.51 (0.32, 7.15)	0.605	1.46 (0.27, 7.81)	0.659
**Ethnicity**				
Caucasian	Ref		Ref	
Indigenous	3.27 (1.93, 5.53)	**< 0.001**	2.46 (1.35, 4.49)	**0.003**
Asian/African/Black/ Caribbean/Latin	0.65 (0.23, 1.90)	0.435	0.83 (0.28, 2.48)	0.744
Other	2.02 (0.94, 4.32)	0.070	1.82 (0.81, 4.08)	0.145
**Employment status**				
Working	Ref			
Unemployed	2.50 (1.49, 4.20)	**0.001**	Not selected^a^	
Other	1.10 (0.57, 2.13)	0.780		
**Incarceration ever**				
No Yes	Ref 3.26 (2.09, 5.09)	**< 0.001**	Not selected^a^	
**Homelessness in the last 12 months**				
No Yes	Ref 3.24 (1.92, 5.49)	**< 0.001**	Ref 2.21 (1.25, 3.90)	**0.006**
**Mental health diagnosis ever**				
No Yes	Ref 1.01 (0.64, 1.61)	0.958	Not selected^a^	
**Sexual activity in the last 12 months** (n = 590)				
No Yes	Ref 0.98 (0.61, 1.55)	0.913	Ref 1.49 (0.89, 2.50)	**0.131**
**History of IDU**				
Never	Ref		Ref	
Yes but more than a year ago	2.05 (1.18, 3.57)	**0.011**	1.78 (0.98, 3.23)	0.060
Yes in the past year	3.72 (2.21, 6.26)	**< 0.001**	2.53 (1.43, 4.50)	**0.002**
**Years of follow-up**	1.14 (0.87, 1.51)	0.351	1.07 (0.80, 1.43)	0.666

Bold text refers to p-values < 0.05

CI: confidence interval

IDU: injection drug use

^a^Confounders were selected for inclusion in the final model using a backward stepwise selection approach, which used the relative change in coefficients of the MOS-SSS score as a criterion, until the minimum change from the full model exceeded 5%

^b^MOS-SSS odds ratio and adjusted odds ratio reported per 10 unit increase. Reference category for TI: ‘No’

## Discussion

In our sample of PLWH across BC who were engaged in care at enrolment, we found that several key sociodemographic variables were correlated with greater social support. Those who were not in a relationship, were diagnosed with a mental health disorder, or who reported experiencing lifetime violence had lower measures of social support. In contrast, those who self-identified as Indigenous, reported a past history of IDU, and those who reported sexual activity in the last 12 months, had higher social support scores. Furthermore, we found that those reporting higher levels of social support were less likely to interrupt their HIV treatment during follow-up. This suggests that social support is an important determinant of treatment engagement and that interventions to improve social support for PLWH may be an effective means of assisting them to remain engaged in care.

A review of antiretroviral therapy adherence interventions published between 1996 and 2004 found that although there has been an increase in interventions targeting ART adherence, there continues to be variability in the efficacy of these interventions [79]. They found that most interventions have small effects but targeted interventions based on participant needs had greater effects on adherence than generalized adherence support [79, 80]. Behavioural interventions that have had greater success were found to use a multidisciplinary approach that utilized several strategies from patient-centered, therapeutic clinical approaches including tailoring the regimen schedule to the individual, having a support person to assist with adherence, assessing adherence in routine clinical care, and providing regular case management [81–83]. Continued research is still needed to identify effective components that can be implemented across settings.

Our finding that individuals who have been diagnosed with a mental health disorder, including depression, anxiety, and bipolar disorder, reported lower levels of social support is also supported in other settings [18, 23–25, 84, 85]. The relationship between mental health and social support have been further explored in other studies that found diagnosed depression, anxiety, positive states of mind, and favourable mental health states act as mediators in the relationship between social support and medication adherence [19, 26, 86] in addition to being a covariate [18, 23, 24]. This may warrant further investigation into the role of mental health in social support and potential impacts on overall health care outcomes. We also found those who had past history of IDU but no recent injection substance use in our sample had significant positive associations with social support in our multivariable analysis. Past research has shown that among PWID with a past history of IDU, those who had greater social support used drugs less frequently in recent months [87, 88], and had greater engagement with HIV medical care [89, 90].

We found the largest differences in median MOS-SSS scores between those who were in a stable relationship (married, common-law or steady partner) compared with those who were not. This association has been demonstrated in previous research, as being in a relationship may provide participants with support that encompasses similar constructs measured in the MOS-SSS scale. Development and validation of the MOS-SSS scale found indicators of marital status moderately correlated with general social support [22, 64, 91]. Constructs found in formal partnerships may similarly be reflected in informal relationships for those reporting sexual activity in the last 12 months [23, 92], with previous studies having reported participants included sex partners as sources of social support in their analyses [89, 93].

In our sample, we found that although those who self-identified as Indigenous were more likely to report higher levels of social support, they were also more likely to experience one or more treatment interruptions. This contradictory finding may be a result of numerous factors related to socio-cultural structures, Canada’s history of colonization, and experiences of racism and discrimination against Indigenous peoples within the healthcare system. This is evidenced in the “In Plain Sight: Addressing Indigenous-specific Racism and Discrimination in BC Health Care” report that documented instances of widespread systemic racism against Indigenous peoples within the BC health care system, contributing to mistrust and avoidance of health services [94]. Previous studies examining HIV care experiences among Indigenous people have further found that a range of risk factors such as homelessness, food insecurity, substance use and mental health issues negatively impact ART adherence [95, 96]. These factors, combined with general mistrust towards the healthcare system may exacerbate the risk of experiencing a treatment interruption among Indigenous people living with HIV, regardless of social support levels [97]. Our findings may suggest that the protective effect of higher levels of social support within Indigenous cultures [98–100] may not be enough to offset the negative impacts resulting from colonization and socio-structural adherence risk factors experienced among Indigenous peoples. Recommendations in the “In Plain Sight” report and the “Truth and Reconciliation Commission of Canada” report [101] outline calls to action to redress these inequalities, including implementing anti-racism policies, along with supporting cultural safety training for health care staff so that Indigenous peoples can feel safe and supported when accessing health care in Canada.

Our findings regarding social support and TIs echo past research in other settings in North America, that have found similar associations between higher social support and adherence to ART [17, 24, 25, 27, 102]. Social support was found to be associated with ART adherence even after controlling for age and alcohol consumption [25], and among a cohort of newly diagnosed individuals, higher social support was associated with earlier HIV diagnosis, timely linkage to care, and ART adherence [17]. More specifically, perceived emotional support and tangible social support were associated with adherence in a cohort of women across the United States [102]. Further, a meta-analysis of patient adherence to medication across a wide range of diseases and treatment regimens found that higher levels of functional social support were more highly related to self-reported adherence while taking into account seriousness of disease, and regimen type [103]. A caveat, however, is this meta-analysis excluded patients on psychiatric treatment regimens, individuals experiencing homelessness, and patients who reported alcohol or drug abuse, which limits the applicability of these findings to our study where participants tend to be structurally marginalized due to HIV status, housing, or substance use.

This study has several strengths as well as some limitations. Our findings arise from a setting where ART is universally available to all medically eligible individuals in the province, with a majority of our sample already demonstrating high levels of engagement in care (90% of participants having no detectable viral load for ≥ 3 months in the year prior to enrolment) [60]. Further, our sample may be unable to capture experiences of those who are truly disconnected from care as we recruited participants from clinics, ASOs, and pharmacies where individuals may already have some degree of engagement in care. A strength of our study is its socio-demographically representative sample of PLWH in the province, with proportional representation by age, gender, key populations, and geography, that reflects the diverse experiences of PLWH across BC [60]. Additionally, with our data linkages to the BC-CfE DTP, we were able to use an objective measure of TIs based on pharmacy refill data, rather than relying on participant self-report. However, as we defined TI as not receiving ART for 90 days or more based on prescription refill data, our findings may underestimate treatment interruptions. Other definitions of TIs such as self-report may also underestimate treatment interruptions but may further be affected by social desirability bias. Despite a more conservative estimate of TI experiences, we still found an association between social support and TI. We also used a validated measure of social support, the MOS-SSS, which has previously been shown to have good reliability with a high Cronbach’s alpha (over 0.90), in patients with chronic health conditions, and amongst older adults in Canada [22, 64, 65].

## Conclusions

In our cohort of PLWH in BC, we found that greater social support was significantly associated with lower odds of TIs. This suggests that connectedness to others, and those who have someone they can regularly rely on, have an important role in positively impacting treatment engagement and health outcomes of PLWH. Our findings on the impacts of social support on TIs warrant further examination of strategies and programs that promote HIV medication adherence particularly among those with lower social support including those diagnosed with a mental health disorder. A review of clinical and behavioural programs emphasizing increasing social supports have identified promising strategies that may promote treatment engagement [79–83]. Future work should examine effective means to build social support among communities using practice-based evidence, and potentially integrating social support structures into HIV clinical practice.

### Electronic supplementary material

Below is the link to the electronic supplementary material.

**Supplementary Material 1**: Medical Outcomes Study-Social Support Survey (MOS-SSS) Scale Items

## Data Availability

The British Columbia Centre for Excellence in HIV/AIDS (BC-CfE) is prohibited from making individual-level data available publicly due to provisions in our service contracts, institutional policy, and ethical requirements. In order to facilitate research, we make such data available via data access requests. Some BC-CfE data is not available externally due to prohibitions in service contracts with our funders or data providers. For more information or to make a request, please contact Mark Helberg, Senior Director, Internal and External Relations, and Strategic Development: mhelberg@bccfe.ca. All data related to this manuscript are provided in the main body of the paper and Supporting Information files.

## List of abbreviations

ART: Antiretroviral therapy
ASOs: AIDS Service Organizations
BC-CfE: British Columbia Centre for Excellence in HIV/AIDS
BC: British Columbia
DTP: BC-CfE Drug Treatment Program
gbMSM: gay, bisexual, or other men who have sex with men
IDU: Injection drug use
MOS-SSS: Medical Outcomes Study – Social Support Survey
PLWH: People living with HIV
PRA: Peer research associate
PWID: People who use injection drugs
SHAPE: STOP HIV/AIDS Program Evaluation study
TasP: Treatment as Prevention
TI: Treatment interruption
