# Supplementary material for: It’s all about connection: Determinants of social support and the influence on HIV treatment interruptions among people living with HIV in British Columbia, Canada
Source: BMC Public Health. 2023 Dec 16;23:2524. doi: 10.1186/s12889-023-17416-7 (PMC10725596; doi:10.1186/s12889-023-17416-7)
Supplement: Supplementary file 1 — Supplementary Material 1: Medical Outcomes Study-Social Support Survey (MOS-SSS) Scale Items [file 12889_2023_17416_MOESM1_ESM.doc]

Appendix. Medical Outcomes Study – Social Support Survey: Scale Items (Sherbourne et al., 1991)

People sometimes look to others for companionship, assistance, or other types of support. How often is each of the following kinds of support available to you if you need it?

|  | **None of the time** | **A little of the time** | **Some of the time** | **Most of the time** | **All of the time** |
| --- | --- | --- | --- | --- | --- |
| 1. Someone to help you if you were confined to bed |  |  |  |  |  |
| 2. Someone you can count on to listen to you when you need to talk |  |  |  |  |  |
| 3. Someone to give you good advice about a crisis |  |  |  |  |  |
| 4. Someone to take you to the doctor if you needed it |  |  |  |  |  |
| 5. Someone who shows you love and affection |  |  |  |  |  |
| 6. Someone to have a good time with |  |  |  |  |  |
| 7. Someone to give you information to help you understand a situation |  |  |  |  |  |
| 8. Someone to confide in or talk to about yourself or your problems |  |  |  |  |  |
| 9. Someone who hugs you |  |  |  |  |  |
| 10. Someone to get together with for relaxation |  |  |  |  |  |
| 11. Someone to prepare your meals if you were unable to do it yourself |  |  |  |  |  |
| 12. Someone whose advice you really want |  |  |  |  |  |
| 13. Someone to do things with to help you get your mind off things |  |  |  |  |  |
| 14. Someone to help with daily chores if you were sick |  |  |  |  |  |
| 15. Someone to share your most private worries and fears with |  |  |  |  |  |
| 16. Someone to turn to for suggestions about how to deal with a personal problem |  |  |  |  |  |
| 17. Someone to do something enjoyable with |  |  |  |  |  |
| 18. Someone who understands your problem |  |  |  |  |  |
| 19. Someone to love and make you feel wanted |  |  |  |  |  |
